# DEM calibration insights on the role of particle shape for sub 2 mm particles

**DOI:** 10.1038/s41598-025-04592-2

**Published:** 2025-07-01

**Authors:** Jan Diviš, David Žurovec, Álvaro Ramírez-Gómez, Jakub Hlosta, Jiří Rozbroj, Kamila Pokorná, Jan Nečas, Jiří Zegzulka

**Affiliations:** 1 Department of Mining Engineering and Safety, Faculty of Mining and Geology, VSB-TU Ostrava, 17. listopadu 15/2172, 708 33 Ostrava-Poruba, Czech Republic; 2 Department of Mechanical, Chemical and Industrial Design Engineering, Universidad Politécnica de Madrid, Ronda de Valencia 3, 28012 Madrid, Spain

**Keywords:** Discrete element method, Calibration technique, Polyhedral particles, Non-spherical particles, Sphere, Computational science, Engineering

## Abstract

This study introduces a comprehensive calibration technique for discrete element method (DEM) simulations. Its focus is on particles smaller than 2 mm and this showcase shows comparison between spherical and polyhedral particle shape calibration. Very fine powders or particulate materials with small particles are usually calibrated with upscaling. Unfortunately, some applications are dependent heavily on a quite precise particle size range, such as abrasion, crushing, pneumatic conveying, feeding, and dosing. Traditional DEM simulations often rely on spherical or multi-spherical particle models, which lack the precision needed, particularly due to the surface waviness introduced in the latter case. This limitation impacts dynamic industrial applications like mixing, hopper discharge, and abrasion. To address this gap, we present a comparative calibration approach for spherical and polyhedral particles, using silica sand as the test material. The calibration combines static and dynamic parameters such as rolling resistance, particle-to-particle restitution, and wall friction, validated through experiments on a powder flow calibration stand. Results revealed significant differences in flow dynamics, highlighting the enhanced realism of polyhedral models despite increased computational demands. This work provides a comprehensive framework for DEM calibration of fine particulate materials, specifically validated for particle sizes between 400 and 1500 µm, improving simulation accuracy and extending applicability across various industrial processes.

## Introduction

Discrete element method (DEM) is a powerful numerical technique used to simulate the behaviour of granular materials, such as powders, grains, pellets etc.^1^. DEM is particularly well-suited for studying complex interactions and dynamic behaviour within particle assemblies. Recent advancements in DEM have focused on developing models with non-spherical particles, aiming to better capture the realistic behaviour of particulate systems. The effectiveness of modern computational models relies on accurately and efficiently accounting for particle shape^2^. To represent the particle shape different approaches have been used such as ellipsoid^3^, multi-sphere^4^, super-ellipsoid^5^, bonded-sphere^6^, and polyhedron^7^. DEM with polyhedron particles is increasingly recognized as a valuable tool for simulating and understanding the behaviour of particulate systems across a wide range of applications, offering enhanced realism and predictive capabilities compared to traditional spherical particle models.

Polyhedral particles are found in geotechnical engineering^8^, civil engineering^9^, mining and mineral processing^10^, and process engineering^11^. The simulations using polyhedral particles have made significant progress evolving from showing on how contacts between the polyhedral particles function^12^, to optimizing a polyhedral particle meshes^13^, improving particle–particle contact algorithms^14^, and conducting predictive studies across various industries^11^. Recent studies have emphasized the significance of particle shape with multi-level morphology^15,16^ showing that each morphological factor contributes differently to macro- and micro-mechanical responses and DEM simulations yield more consistent results with physical experiments, particularly in capturing phenomena such as interlocking, rolling resistance, and force chain propagation^17,18^.

The DEM calibration of the particulate material behaviour is inherently complex as each input parameter influences the material’s behaviour. Achieving a suitable combination of parameters, particularly those that dominate in a given application, is crucial^19^. Nowadays, the most accurate DEM calibrations involve sets of carefully designed calibration experiments. The most common experiments used in these sets are packing tests^20^, and pilling or pouring tests^21^. Further, simulations are validated by hopper discharge tests or rotational drum tests^22,23^. Attempts have been made to standardise calibration processes^24^. Another improvement was introduced by Richter^25^, who presented an algorithm to find the most optimal combinations of input parameters for DEM simulations. An example on how to deal with calibrations is given by Lu^26^, who took calibrated input parameters from previous work.

However, most of these calibration sets are calibrated with spherical or multi-sphere particles^27^, not with polyhedral particles. Furthermore, the calibrations of models with polyhedral particles are often insufficient, which reduces the credibility of subsequent simulation studies. Some simulation studies, such as^12,28^, were conducted without experimental calibration; however, these works primarily aimed to introduce mathematical frameworks for polyhedral contact detection and overlap evaluation rather than replicate real physical systems.

Liu^29^ developed comparison of polyhedral shapes coupling discrete element modelling (DEM) and computational fluid dynamics (CFD). Liu used 3600 particles with the smallest particle dimension of 4 mm. Simulations were calibrated by initially filling the bed and the validated through the fluidization of the packed bed. Puga^30^ conducted study of polyhedral particles in a rotating drum. He used particles larger than 25 mm, the simulation time step was in a range of 1e10^−4^ to 1e10^−5^, and the maximum number of particles in the simulation did not exceed 1100. Polyhedral particles have a high potential, especially due to their shape, which enables highly accurate collision behaviour and more realistic particle interlocking. Their geometry can be scanned through different scanning techniques available and precisely replicated in DEM software^31^. However, this high level of accuracy comes at the cost of increased computation times. Despite this, the calibration of polyhedral particles will be necessary because nowadays, advanced breakage models^32^ capable of fragmenting spherical and ellipsoidal particles into polyhedral fragments^33^, which further supports the need for robust DEM calibration methods involving polyhedral shapes. As these models increasingly rely on accurate representation of breakage products, the calibration of polyhedral particles becomes essential for reliable simulation of breakage processes. A partial solution to reducing the computation time for polyhedral particles was shown by Illana^34^. Illana showed the application of zones where replacing polyhedral particles with spherical particles can reduce computational time while preserving the benefits of polyhedral behaviour in zones where it is important. Kwon^35^ tried to minimize computational demands by comparing 2D and 3D simulations of rod-shaped particles discharge from a hopper. Similarly, Mack^36^ conducted studies using a mixture of 322 polyhedral dice of various shapes, including hexahedron, octahedron, icosahedron, pentagonal trapezohedron and tetrahedron, with particles exceeding 13 mm. Park^37^ demonstrated computational studies that using special algorithms could significantly improve the performance of simulations involving millions of polyhedral particles. Jadidi^38^ calibrated cubes and cylindrical particles in a double paddle blender during mixing, using over 95,000 particles with a minimum dimension of 6 mm. Liu^11^ performed a comparative study on the calibration of polyhedral and spherical particles (multi-sphere, multi-super-ellipsoid). In this study, Liu calibrated 800 polyhedral particles by packing them in a cylinder and through piling experiments, validating particle flow in a rotating drum with up to 1800 particles. Further studies with 10,000 particles in a rotating drum showed that polyhedral particles provided the best match for table flow, comparing with the results obtained from multi-spheres and multi-super-ellipsoids models.

In summary, most studies involving polyhedral particles use particles larger than 10 mm, use a number of particles smaller than a few thousands, use a single large polyhedral particle, or are studies conducted without calibration and/or validation of the DEM model. There is a gap in calibration and validation for the simulation of dynamic applications where calibration by pilling experiment is not sufficient. Although DEM calibration using the rotating drum experiment is very well fitted for applications such as mixing^39^ and tablet coating^40^, there are applications for which a calibration using rotating drum experiment may not accurately represent the dynamic behaviour of the particulate material, such as crushing^10^, grinding^41^, thermal conductivity of the particulate material^42^, bin or hopper discharge^43^, feeding^44^, segregation^45^, abrasion^46^, erosion^46^, or in a coupling simulations such as DEM-CFD^29,47^, and DEM-SPH^47,48^.

Therefore, we present the calibration method that is done for over 100,000 spherical and polyhedral particles smaller than 2 mm using dynamic particle behaviour. The study specifically targets non-cohesive, frictionally interacting particles in the 400–1500 μm size range. Cohesive forces and surface energy effects were not considered in this work, as the particle sizes are above the typical threshold where adhesion dominates. The calibration method is comparable with Roessler’s calibration method^24^, but it incorporates its own modifications. Conducting a comparative study of spherical and polyhedral particles in DEM calibration for non-cohesive particulate materials is essential to improve model accuracy, enhancing predictive capabilities, optimizing DEM parameters, and advancing fundamental knowledge across various industries and scientific disciplines. This study highlights computational differences and significantly influence of particle shape on DEM calibration accuracy.

## Materials and methods

### Particulate material

The material chosen for this study was silica sand. The particulate material was supplied by Bonatrans Group a.s. (Bohumín, Czech Republic). The decisive factors were primarily the shape, size, and hardness of the particles, but also their non-cohesiveness and good flowability of the particulate material.

### Particle shape measurement

**Fig. 1:**
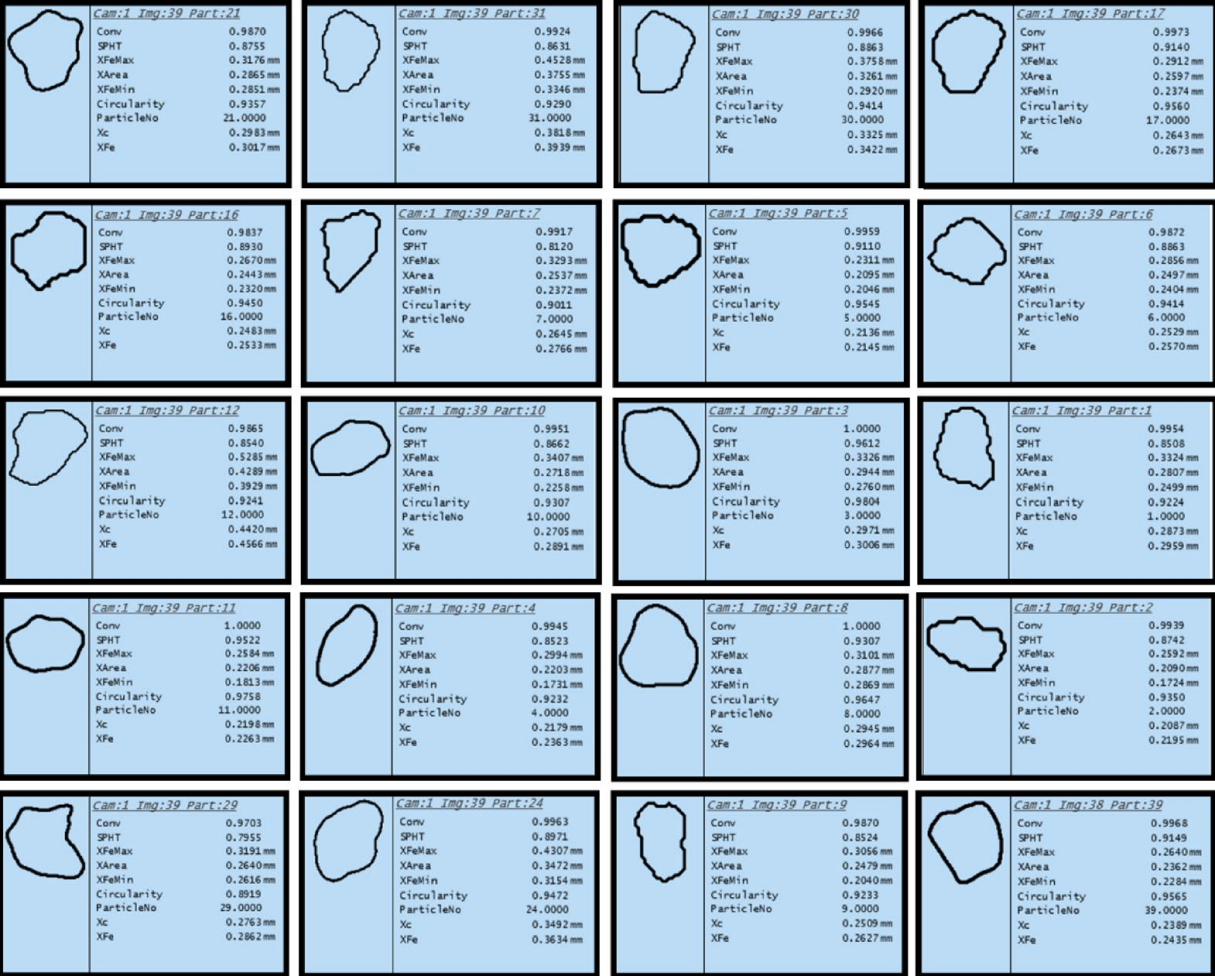
Shape parameters obtained with the CAMSIZER 3D.

The CAMSIZER 3D (Microtrac Retsch GmbH, Germany) was used to characterise morphological features of particulate materials, with a measurable particle size range of 20–3000 μm. The CAMSIZER 3D was used for the quantification of the particle shape as well. The shape parameters were measured by two cameras, which operate on the principle of capturing particles in free fall through a sensing zone. Particles are fed into this zone by a vibrating feeder. The Camsizer 3D software records the length, width, and thickness of each particle and also evaluates other shape parameters (Fig. 1)^49^. This method eliminates the need for subjective image editing to eliminate overlapping particles. In this study, only the aspect ratio of the particles was used to determine the horizontal and vertical dimensions of the polyhedral particles.

### Virtual particle shape

The particle shape is an input parameter, which, according to Lu et al.^50^, is considered one of the most important input parameters in DEM modeling. Its accuracy is crucial for the model to be sufficiently predictive. In most DEM simulations, spherical particles are still preferred. When using spherical particles, the strength of the particle system is lower compared to the actual frictional strength of particulate materials with non-spherical particles. Shear strength is most commonly increased by using rolling friction, which limits particle rotation^51,52^. Nowadays, non-spherical particles are approximated using ellipsoids, superquadrics, polyhedrons, and clusters (spherical or composite clusters).

To compare different virtual particle shapes, we chose spherical and polyhedral particles as representative models. The virtual spherical particles of silica sand were calibrated using input parameters that modify their behavior to mimic the characteristics of non-spherical particles.

### Particle size distribution measurement

Particle size distribution is also considered as an input parameter and should be carefully determined. When the particle size distribution is measured in a laboratory setting, it is possible to model particle sizes accurately. However, in real industrial applications, it is often not feasible to model the full scale due to the computational power limitations. In industrial conditions, millions^53^, billions^54^, or even trillions^54^ of particles can be found. To address this, methods such as particle upscaling are used, where particle size is increased while maintaining the size of the simulated domain^55^. Additionally, particles smaller than a certain size may be ignored^53^, or smaller particles may be scaled up^56^. Roessler and Katterfeld^53^ noted that when particles are upscaled, the overall degrees of freedom in the particle system decrease. This prevents the use of the same input parameter values as those for the original particles, because bulk behavior depends on the total number of interactions, such as the number of contacts and degrees of freedom. The input interaction parameters must therefore compensate for the absence of smaller particles, which is achieved through calibration^54^.

In the Rocky DEM simulation environment, particle size distribution can be specified. The particle size distribution for silica sand, was determined using the Mastersizer 3000 laser diffraction analyzer (Malvern Panalytical Ltd, United Kingdom). Using the dry method of the Mastersizer analyzer, data were collected in the range from 0.01 to 3500 μm. The sample was analyzed in three repetitions. The Fraunhofer diffraction theory was used to determine particle size based on the light intensity distribution pattern. Average values were calculated using the Mastersizer 3000 software^57^.

### Powder flow calibration (PFC) stand: setup and measured parameters

**Fig. 2:**
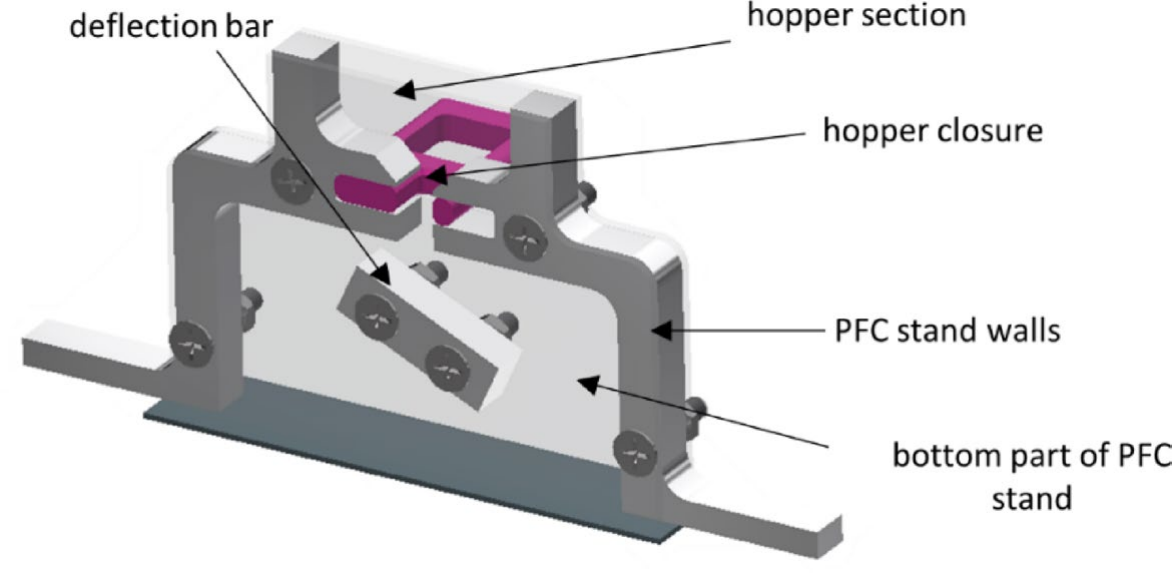
3D design of the particle flow calibration (PFC) stand.

**Fig. 3:**
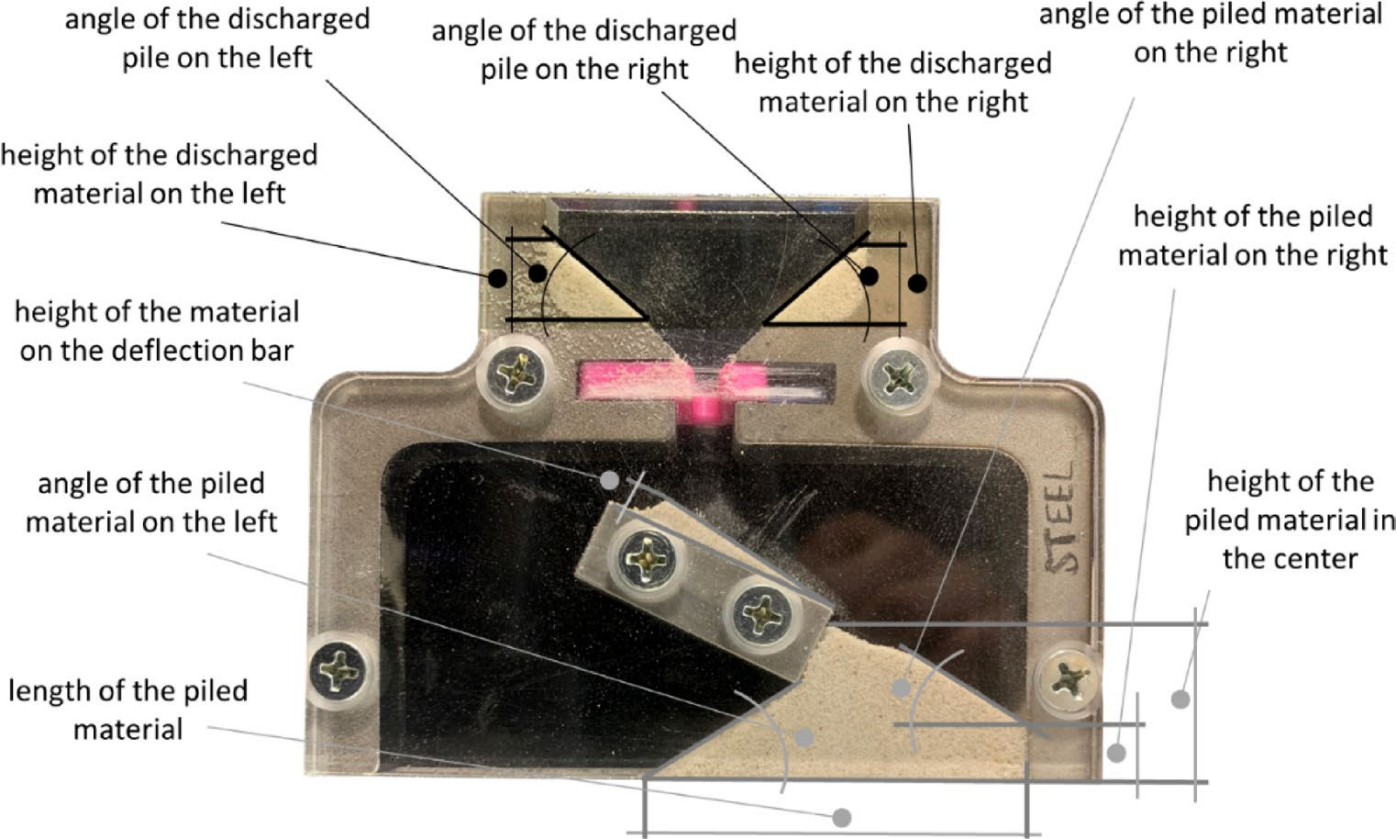
The experimental evaluation of individual parameters obtained from the powder flow calibration (PFC) stand.

In this study, a method of experimental measurement for the particulate material behavior was chosen that combines several of the normally used methods. This approach is rarely used because it is extremely time-consuming to align all parameters given the large number of combinations offered by the input parameters. The selected method combines material pouring into a hopper (volume filling calibration), emptying the hopper (validation of the calibration simulation using emptying time), material discharge over an obstacle (dynamic behavior calibration), the static angle of repose after hopper emptying (discharged angle of repose calibration), and the static angle of repose after bouncing off the obstacle (poured angle of repose calibration). Figure 2 illustrates the experimental powder flow calibration (PFC) stand. In total, 9 parameters were tracked during the calibration simulations, all of which needed to be calibrated to either match or closely approximate experimental measurements. Figure 3 illustrates the experimental evaluation of individual parameters obtained from the powder flow calibration (PFC) stand.

The PFC stand was designed for particulate materials with particles smaller than 2 mm. The presented calibration method is currently applicable for particle sizes in the range of approximately 50–2000 μm, with the lower limit defined by limitations in material handling, camera resolution, and particle inertia during free fall. Below this range, measurement errors and cohesive effects become significant. Above this range, particle scale begins to interfere with experimental apparatus dimensions. A precise analysis of lower and upper boundaries is the subject of future work. Given the computational time and the large number of simulations, the smallest suitable particle size considered in this research work ranged from 200 to 400 μm.

### Wall friction of the particulate material

**Fig. 4:**
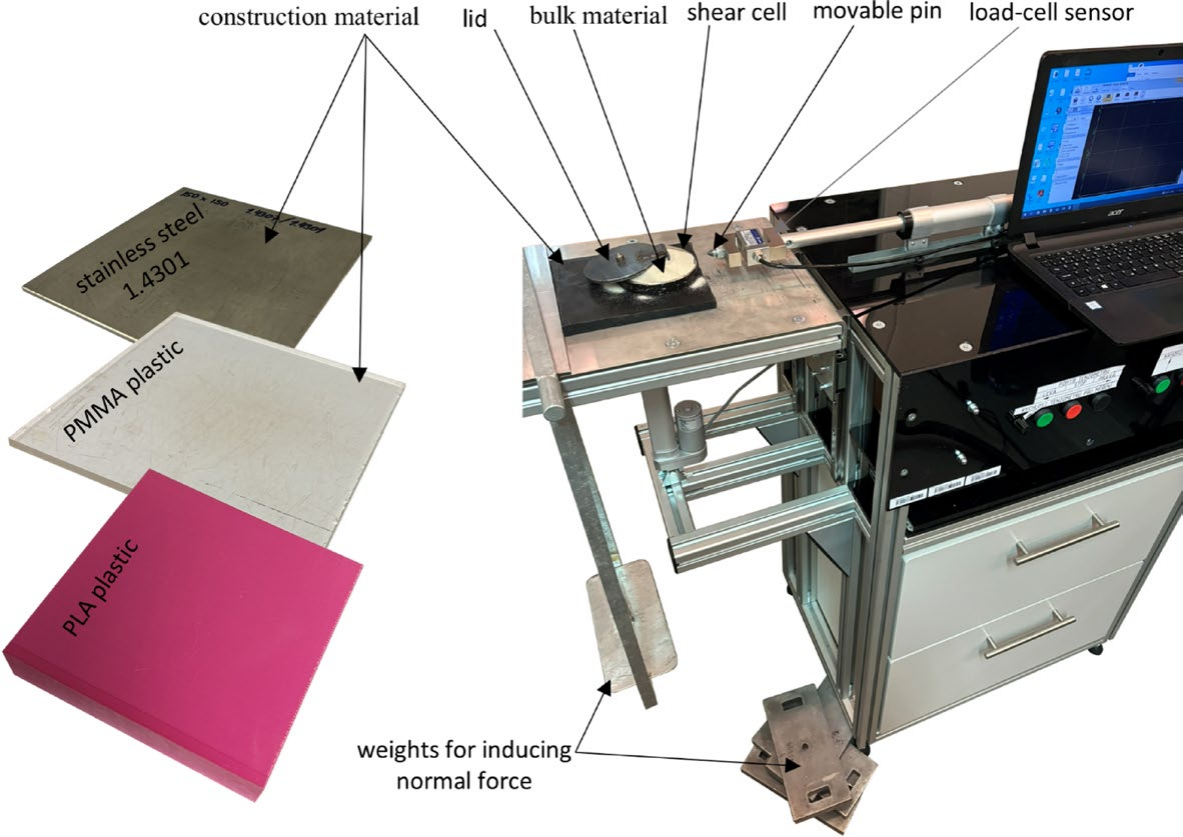
Jenike direct shear tester for measuring the wall friction angle.

The frictional parameters between particulate material and construction materials were measured using a Jenike direct shear tester (Fig. 4). This device determines the relationship between the shear force (*FS*) and the normal load (*FN*). The shear force is required to move the particulate material over the construction material under a normal load. A movable pin applies the shear force, which is detected by the deformation of a strain gauge located before the pin. This frictional parameter, also referred to as wall friction, represents the energy loss when the particulate material slides over the construction material^58^. The construction materials used in this study were selected based on their application in calibration experiments and DEM calibrations. The construction materials included were stainless steel (1.4301), PMMA and PLA plastic.

Stainless steel 1.4301 was used to represent all steel materials, as steel typically exhibits similar external friction characteristics, with the final surface treatment or wear playing a much more significant role in determining differences. This simplification was adopted because Jenike’s measurements focus on a set of particles, while DEM interaction parameters are defined for each particle. The external friction coefficient (*μ*_*W*_) was used as the input for both static (*SF*) and dynamic friction (*DF*) parameters, serving as a baseline value that can be adjusted during calibration to ensure the virtual particulate material matches the behavior observed in experiments.

### Coefficient of restitution particle-construction material ***e***_***pw***_

“The coefficient of restitution is the ratio of the final to the initial relative velocity of colliding objects. This ratio typically ranges from 0 to 1, where 0 represents a perfectly inelastic collision and 1 represents a perfectly elastic collision”^59^.

The coefficient of restitution between a particle and a construction material *e*_*pw*_ is most commonly measured using a gravitational method^21^. A particle is dropped from a certain height onto the surface of the construction material, and the amount of energy absorbed during impact is analyzed. By comparing the kinetic energy just before *E*_*k1*_ and after impact *E*_*k2*_, the coefficient of restitution between the particle and the construction material *e*_*pw*_ can be determined as shown in Eq. (1)^21^.

1 $$e_{pw} = \sqrt {\frac{{E_{{K2 \left( {after\,impact} \right)}} }}{{E_{{K1 \left( {before\,impact} \right)}} }}} = \sqrt {\frac{{\frac{1}{2}{\text{m}} \cdot {\text{v}}_{2}^{2} }}{{\frac{1}{2}{\text{m}} \cdot {\text{v}}_{1}^{2} }}} = \sqrt {\frac{{{\text{v}}_{2}^{2} }}{{{\text{v}}_{1}^{2} }}} = \frac{{v_{2} }}{{v_{1} }}$$

where *m* is particle mass, *v*_1_ is a speed of the particle before the impact, and *v*_2_ is a speed of the particle after the impact.

In the case of a falling object, initially at rest, dropped from a height *h*_1_ onto a surface made of construction material, we can compare the potential energies *E*_*P*_. From this assumption, it follows that at the moment of impact, the particle has zero velocity, and after bouncing back to the rebound height *h*_2_, it again reaches zero velocity before falling once more. At all three points (before the fall, after the impact, and at the peak of the rebound), the kinetic energy *E*_*k*_ is zero. The coefficient of restitution based on potential energy is expressed by Eq. (2)^21^.

2 $$e_{pw} = \sqrt {\frac{{E_{{P2 \left( {rebound\;heigth} \right)}} }}{{E_{{P1 \left( {impact\;heigth} \right)}} }}} = \sqrt {\frac{{{\text{m}} \cdot {\text{g}} \cdot {\text{h}}_{2} }}{{{\text{m}} \cdot {\text{g}} \cdot {\text{h}}_{1} }}} = \sqrt {\frac{{{\text{h}}_{2} }}{{{\text{h}}_{1} }}}$$

**Fig. 5:**
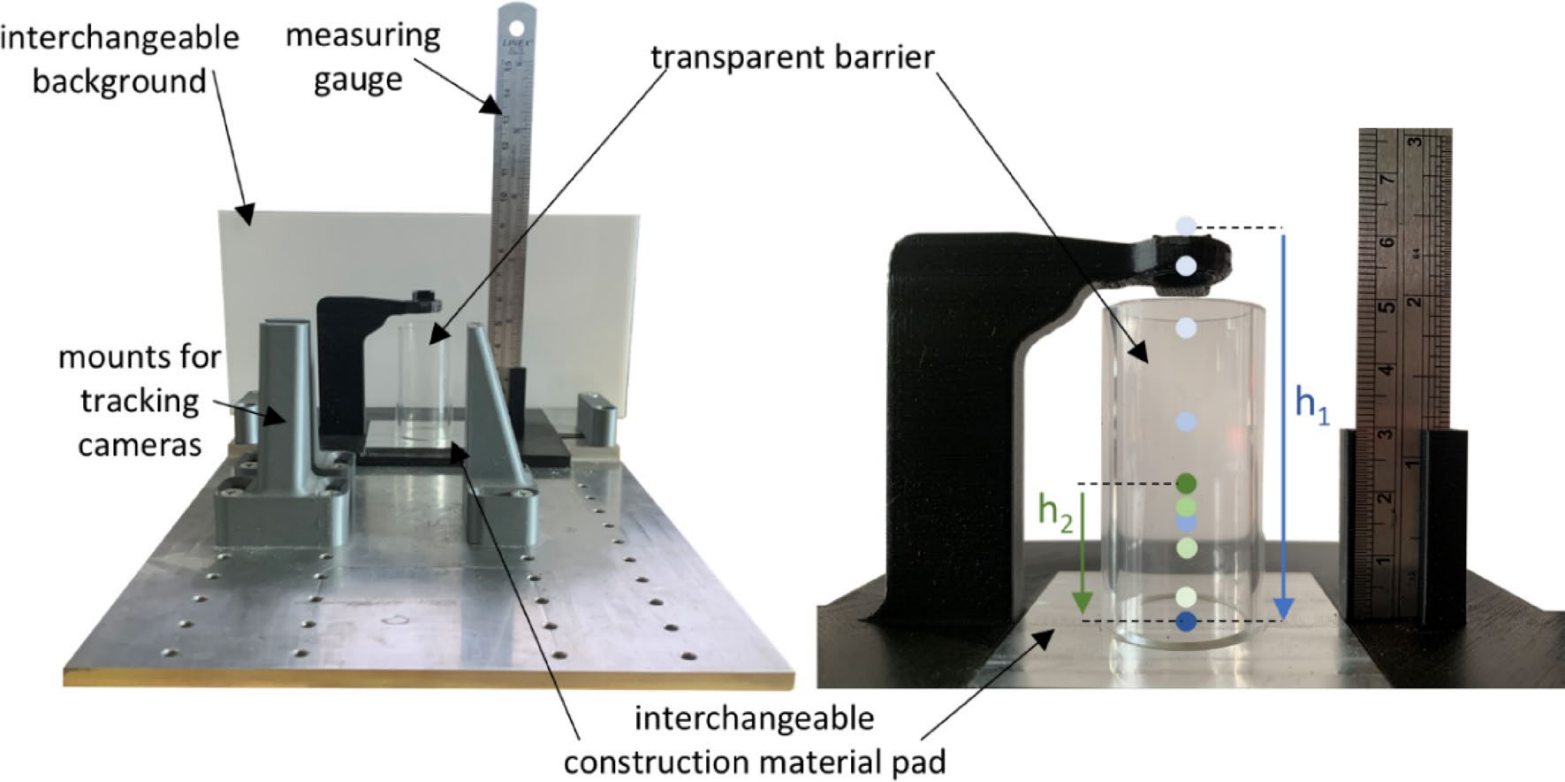
The coefficient of restitution experimental stand.

An experiment setup was used in which a particle was released from rest at a predetermined height. The construction material of the base was varied. Tests for the coefficient of restitution were conducted for the selected construction materials: stainless steel 1.4301, PMMA, and PLA plastic. The experimental apparatus is shown in Fig. 5. The particle is released from a stationary position on the upper platform. As it falls freely, it gains speed and impacts the plate. The entire process, from the particle’s fall from height* h*_1_ to its rebound back to height *h*_2_, is recorded using a high-speed camera or a camera with an enhanced frame rate function (up to 240 fps). This frame rate was sufficient to capture the particle’s rebound height *h*_2_ and determine the ratio between the two heights *h*_1_ and *h*_2_. A total of 20 experimental measurements (20 different particles) were conducted to evaluate the coefficient of restitution between the particulate material particles and the given construction material. To ensure that the particle does not deviate from the vertical plane, the experimental apparatus is equipped with transparent barrier (Fig. 5).

While this setup helps to partially mitigate the effects of particle non-sphericity, it cannot eliminate them entirely. We acknowledge that the rebound-based measurement of the coefficient of restitution is not as precise as it could be, particularly for non-spherical particles. Nevertheless, a representative value was experimentally measured, as DEM software requires a single input for the restitution coefficient—despite the fact that, in reality, this coefficient varies with each collision depending on impact angle, contact geometry, and local conditions. This is precisely why DEM calibration must be performed for the entire particle assembly, rather than relying solely on isolated pairwise measurements. The calibration process inherently compensates for such model simplifications by adjusting interaction parameters to reproduce observed bulk behavior.

### Volume filling experiment

Particulate materials in industry are typically loosely packed, with their arrangement influenced by boundary conditions like hopper walls, partitions, and contact materials. These conditions, along with the material’s intrinsic properties, allow for the study and adjustment of bulk particle arrangement in DEM models through input parameters.

The bulk particle arrangement was analyzed for the entire particulate material set, considering factors such as particle shape, Young’s modulus (*E*), particle mass (*m*_*p*_), and input interaction parameters such as static (*SF*) and dynamic friction (*DF*) (both, between particles and between particle and contact material), rolling resistance (*RR*), and the coefficients of restitution (*e*_*pp*_ and *e*_*pw*_).

The chosen calibration method included filling a fixed space functioning as a hopper (Fig. 2). This space was identical in both the experimental setup and virtual calibration simulation. In the experimental phase, the mass required to fill the hopper section with the given particulate material was measured.

### Calibrated input parameters of the DEM calibrations

Comprehensive calibration simulations were conducted to analyse how virtual particulate matter behavior changes with different input parameters. These simulations focused on the particle-to-particle restitution coefficient *e*_*pp*_, the static (*SF*) and dynamic (*DF*) friction coefficients between particles, and the rolling resistance coefficient (*RR*). This approach involved systematically varying these key parameters. The restitution coefficient *e*_*pp*_ ranged from 0.1 to 0.9 in increments of 0.1, while the *SF* and *DF* coefficients were kept identical for each simulation, also ranging from 0.1 to 0.9 in 0.1 increments. Although Rocky DEM allows separate definitions for static and dynamic friction, both were assumed equal (*SF* = *DF*) in this study to reduce calibration complexity. This combination of *e*_*pp*_, *SF*, and *DF* produced a total of 81 simulations in a single calibration set. The entire set was then simulated with varying *RR* values.

The virtual silica sand with spherical particles was calibrated using *RR* values of 0.10, 0.15, 0.20, and 0.25. The virtual silica sand with polyhedral particles was calibrated using *RR* values of 0.00, 0.05, and 0.10. The *RR* values for spherical particles were varied in a wider range (0.1–0.25) because spherical particles inherently lack resistance to rotation. Rolling resistance thus acts as a proxy for shape-induced interlocking. In contrast, polyhedral particles exhibit geometric interlocking due to their angular shapes, which naturally restrict rotational motion. Therefore, smaller *RR* values (0.00, 0.05, and 0.10) were sufficient to capture realistic behaviour without artificially amplifying resistance to rotation.

### Rolling resistance coefficient (*RR*)

The rolling resistance coefficient (*RR*) is a key input parameter in DEM simulations^22^. Unlike in engineering practice, where *RR* is typically expressed as a lever arm with units of length, in the Rocky DEM simulation environment, this dimensionless parameter is defined as the tangent of the maximum slope angle at which the moment of *RR* balances the moment induced by the particle’s gravitational force. Rocky DEM allows spherical particles to behave as if they were non-spherical. Additionally, the simulation assumes both particles and construction materials are perfectly rigid^21^.

While rolling resistance (*RR*) is often initially estimated using a tilting test apparatus^21^. The small particle sizes in this study made such experiments challenging due to weight and handling difficulties. Additionally, the behavior of particulate materials in bulk assemblies often differs from that of individual particles. Non-spherical particles, in particular, exhibit a wide range of *RR* values, making it difficult to derive a single representative value for simulations.

As a result, *RR* was calibrated directly within the DEM simulation environment. These considerations led to its inclusion as a critical parameter in the calibration process.

### Coefficient of restitution particle–particle ***e***_***pp***_

The coefficient of restitution between two particles *e*_*pp*_ depends on the conservation of energy, similar to the coefficient of restitution between a particle and a construction material. “More specifically, it relates to the conservation of momentum, where the momentum of an isolated system of bodies is conserved. After the collision of two particles, their resulting movements must satisfy the principle of linear momentum conservation”^60^. If particle *A* has a mass *m*_*A*_ and an initial velocity *v*_*A*1_, and particle *B* has a mass *m*_*B*_ and an initial velocity *v*_*B*1_, then after a collision, the total momentum remains constant, resulting in final velocities *v*_*A*2_ and *v*_*B*2_. This conservation of momentum shown in Eq. (3) can be expressed as:

3 $$m_{A} \cdot \left( {\overrightarrow {{v_{A} }} } \right)_{1} + m_{B} \cdot \left( {\overrightarrow {{v_{B} }} } \right)_{1} = m_{A} \cdot \left( {\overrightarrow {{v_{A} }} } \right)_{2} + m_{B} \cdot \left( {\overrightarrow {{v_{B} }} } \right)_{2}$$

The measurement of the particle-to-particle restitution coefficient *e*_*pp*_ is rarely discussed in the literature due to its complexity, as factors like particle mass, shape, and collision direction affect real experiments. A method involves dropping a particle onto a surface of other particles, but ensuring a consistent rebound direction is difficult and requires many repetitions. More commonly, the coefficient of restitution describes the collision between two moving particles, with *e*_*pp*_ defined as the ratio of relative velocities before and after the collision^60^.

4 $$e_{pp} = \frac{{\left( {v_{B} } \right)_{1} - \left( {v_{A} } \right)_{2} }}{{\left( {v_{A} } \right)_{1} - \left( {v_{B} } \right)_{1} }}$$

Another method for measuring the particle-to-particle restitution coefficient *e*_*pp*_ involves colliding two particles on pendulums^21^. One particle is released from a height and swings down to collide with a stationary particle. The rebound height *h*_2_ is compared to the release height *h*_2_, similar to the particle-to-material restitution experiment. However, this method faces challenges such as off-axis movement, pendulum entanglement, and particle rotation, especially with non-spherical particles, leading to approximate results that require fine-tuning during calibration. Both particles and materials must also be sufficiently rigid for accurate measurements^21^.

In this study, the pendulum method could not be used due to the small particle sizes. Although pendulum experiments were attempted, the influence of the pendulum on small particles skewed the results. Despite the adequate stiffness of the materials, hundreds of tests would have been needed to obtain accurate, undistorted restitution coefficient values for non-spherical particles.

### DEM calibrations setup

The input parameters for the DEM calibrations, such as Young’s modulus *E*, Poisson’s ratio *ν*, and density/bulk density *ρ*/*ρ*_*S*_, were selected^61^. Silica sand’s bulk density parameter was measured, density parameter is calculate by Rocky DEM software using bulk density parameter, and silica sand’s Young’s modulus and Poisson’s ratio were chosen to lower computational demand. The input parameters for the DEM calibrations are provided in Table 1.

**Table 1:** Chosen input parameters of the DEM calibrations.

Input parameter	Notation	Unit	Silica sand	Stainless steel (1.4301)	PMMA	PLA plastic
Density	*ρ*	(kg m^−3^)	2420	7850	1180	1200
Bulk density	*ρ*_*S*_	(kg m^−3^)	1450	–	–	–
Young’s modulus	*E*	(MPa)	0.6	210	70	10
Poisson’s ratio	*ν*	(–)	0.3	0.25	0.3	0.3

The Young’s modulus of silica sand was set to 0.6 MPa for numerical reasons, following common DEM practice. Although the actual modulus of quartz-based sand is typically in the range of 60–70 GPa, using such high stiffness values leads to very small time steps and excessive computational cost. Therefore, a reduced modulus was adopted to improve computational efficiency, as the mechanical stiffness in DEM simulations often plays a secondary role and can be compensated through calibration of contact parameters. Similar reductions have been applied in previous DEM studies^62^.

The virtual silica sand underwent simulated calibrations to adjust its behavior according to experimental observations. In total, 9 parameters were tracked during the calibration simulations. The parameters included those from the upper part of the calibration powder flow calibration (PFC) stand (angle and height of the discharged pile) and those from the lower part (middle and right height of the piled material, left and right angles of the piled material, length of the piled material, and the height of the material on the deflection bar).

The simulations were run with the DEM software ESSS Rocky version 23.1.1 for which the simulation parameters are listed in Table 2. The simulations were calculated using graphical processing unit (GPU) NVIDIA GeForce RTX 3060.

**Table 2:** Input parameters for simulations.

Parameter	Value	Unit	Comment
Coefficient of restitution particle–particle *e*_*pp*_	0.1–0.9	–	Scaled by 0.1
Coefficient of static (and dynamic) friction particle–particle *SF* (*DF*)	0.1–0.9	–	Scaled by 0.1
Rolling resistance *RR*	Spheres: 0.10, 0.15, 0.20, 0.25 Polyhedrons: 0.00, 0.05, 0.10	–	
Simulation time step	3.51 × 10^−6^	s	
Rolling resistance model	Type C		Wensrich and Katterfeld^62^
Normal force model	Hertzian spring-dashpot		Hertz^63^
Tangential force model	Mindlin–Deresiewicz		Mindlin and Deresiewicz^64^

The DEM simulations employed the Hertzian spring-dashpot model for normal contact forces^63^, with viscous damping calibrated using a constant coefficient of restitution (particle-construction material *e*_*pw*_ and particle–particle *e*_*pp*_) at a representative velocity. While this simplification is commonly used in DEM workflows, it does not capture the full velocity dependence of coefficient of restitution^65^. In future studies, implementation of velocity-dependent damping models may improve simulation fidelity, particularly for high-energy impacts.

## Results and discussion

Each calibration simulation began with randomly oriented particle generation. Experiments determined that the hopper section could hold 7 g of silica sand, which was set as the target for calibration simulations on the powder flow calibration (PFC) stand. Computational efficiency varied significantly between spherical and polyhedral particle simulations, with spherical particle calibrations completing in approximately 40–70 min, whereas polyhedral particle calibrations required 7–8 h per simulation. Given that each calibration set consisted of 81 simulations, this disparity in computational demand is particularly critical. A full calibration set for spherical particles could be completed within 2–4 days, while the same process for polyhedral particles extended to 23–32 days. This substantial difference underscores the trade-off between computational feasibility and model accuracy, necessitating careful consideration when selecting particle representation in DEM studies. Contact detection for spheres is computationally more efficient because of their geometry allowing for more simple analytical solutions. However, detecting contacts between polyhedral particles becomes more complex. Further results are detailed in the following subsections.

Simulations carried out revealed remarkable differences between spherical and polyhedral particles, especially in flow dynamics and interlocking. These differences were most pronounced at obstacles during discharge, where polyhedral particle exhibited increased material retention.

### Particle shape

Table 3 provides an overview of the shapes of the virtual particles created. The shape parameters of polyhedral particles in Rocky DEM were selected based on measurements from the Camsizer 3D device.

**Table 3:** Particle shape parameters for virtual particles.

Virtual particle shape	Virtual particle	Particle shape parameters	Particle shape parameters
Sphere		Horizontal ratio	1
		Vertical ratio:	1
		Number of corners	0
		Superquadratic degree	2
Polyhedron		Horizontal ratio	1.4
		Vertical ratio:	1
		Number of corners	10
		Superquadratic degree	2

### Particle size distribution

**Fig. 6:**
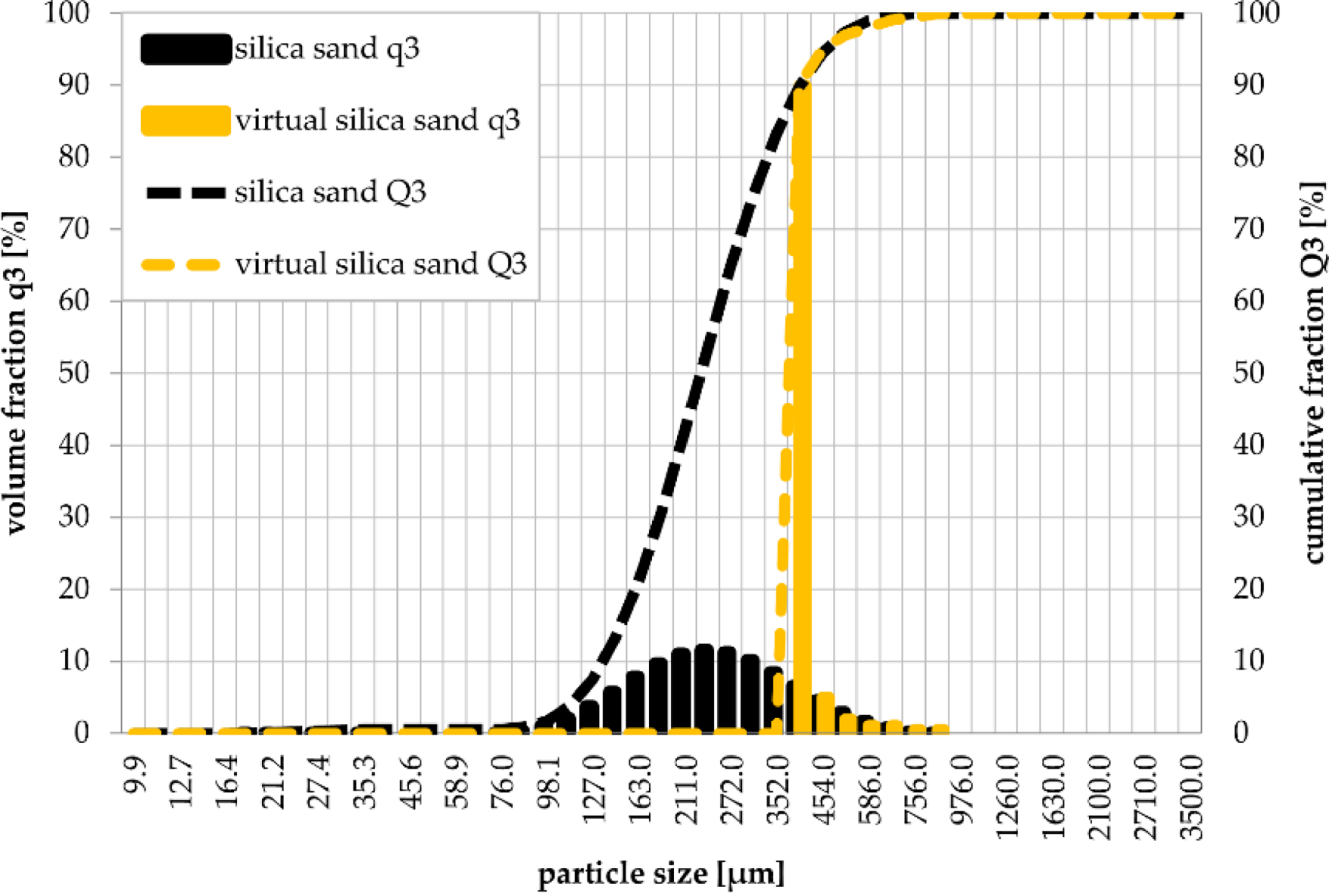
Particle size distributions (volume q3 and cumulative Q3).

Figure 6 provides an overview of the particle size distribution of the silica sand. The statistical parameters d_10_, d_50_, and d_90_ shown in Table 4 are percentile values that indicates the size below which 10%, 50%, and 90% of all particles of the sample are found. These values correspond to the undersieve fractions at each point in the particle size distribution.

**Table 4:** Particle size distribution characterization values d10, d50, and d90.

	d_10_ (µm)	d_50_ (µm)	d_90_ (µm)
Silica sand	152	262	470

The selection of modelled particle size distributions in the DEM software was guided by the available computational resources. In this study, a strategy of excluding smaller particles was implemented to reduce the number of particles in the simulations which therefore decreased computational time. Smaller particles tend to significantly increase contact frequency and collision complexity, making simulations computationally expensive. The chosen sizes for the virtual silica sand particles are listed in Table 5. The particle size distribution curves for both the measured and the selected virtual silica sand are presented in Fig. 6.

**Table 5:** Particle size distribution of the virtual silica sand in DEM software.

Undersieve size (µm)	Cumulative %
860	100
750	99
600	98
450	95
400	90

The presence of fine particles in real material significantly increases the total number of particle-to-particle and particle-to-wall contacts. Removing particles below 0.4 mm reduces these interactions in the simulation, potentially altering bulk behavior, flowability, and shear properties. The deviation between the simulated and measured particle size distribution is acknowledged as a potential source of noise, particularly affecting contact frequency and local porosity. This must be accounted for in the calibration of friction and restitution coefficients. Since smaller particles contribute to increased cohesion, rolling resistance effects, and packing density, their absence in the simulations had to be compensated by adjusting interaction parameters such as wall friction coefficients, rolling resistance, and particle-to-particle friction coefficients. To simplify the system and draw parallels with the use of a single average particle shape, the adoption of a monodisperse particle size distribution could be considered. However, the current study retained a limited polydispersity to better capture packing effects and flow behaviour. Future work will aim to quantify the effect of particle size distribution simplification on simulation accuracy, including comparative studies with monodisperse systems.

### Wall friction of the particulate material

**Fig. 7:**
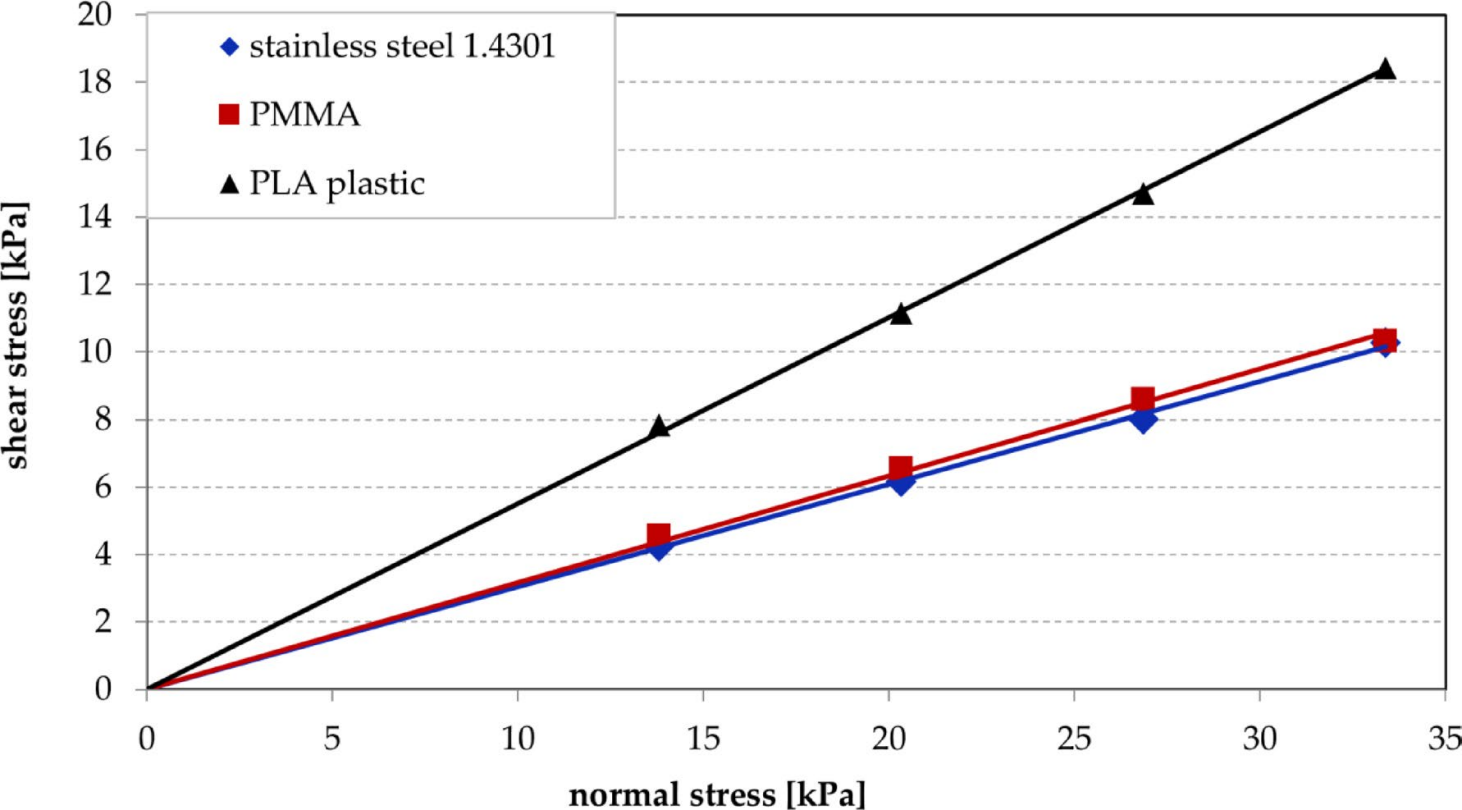
Graph of wall friction curves for silica sand.

Each wall material was tested three times with silica sand, with the average values from these measurements displayed in Table 6. Figure 7 presents the external friction curves for the silica sand. The external friction coefficients were used as input friction parameters for the calibration DEM simulations. These static and dynamic friction parameters (*SF* and *DF*) were entered into the Rocky DEM software to model the interactions between the particles and the wall sample.

**Table 6:** Experimentally measured wall friction of the silica sand.

Wall material	Angle of the wall friction (°)	Standard deviation σ_WFA_ (°)	Coefficient of the wall friction (–)	Standard deviation σ_WFC_ (–)
Stainless steel 1.4301	16.92	0.37	0.30	0.01
PMMA	17.56	1.01	0.32	0.02
PLA	24.76	0.44	0.46	0.01

### Coefficient of restitution particle-to-wall ***e***_***pw***_

In this research, an experimental method was used where a particle was released at a predetermined height. To evaluate the coefficient of restitution *e*_*pw*_ between the particles and the given construction material of the wall sample, 20 repetitions were conducted. The values presented in Table 7 are the average values from these repetitions, calculated according to Eq. (2). Similar results were obtained in the three cases.

**Table 7:** Coefficients of restitution e_pw_.

Wall material	Coefficients of restitution *e*_*pw*_ (–)	Standard deviation σ*e*_*pw*_ (–)
Stainless steel 1.4301	0.51	0.07
PMMA	0.48	0.06
PLA	0.52	0.09

### Powder flow calibration (PFC) stand: test results

Each test on the powder flow tester (PFC) stand was repeated 12 times. From the obtained results, average, maximum, and minimum values were calculated. The final values are summarized in Table 8.

**Table 8:** Values of evaluated PFC experiments.

	Unit	Silica sand			
		Average	Minimum	Maximum	Standard deviation
Upper part					
Angle of the discharged pile—left	(°)	39.62	38.18	41.24	0.81
Height of the discharged pile—left	(mm)	8.97	7.47	9.91	0.57
Angle of the discharged pile—right	(°)	39.24	38.21	40.19	0.63
Height of the discharged pile—right	(mm)	8.93	7.98	9.60	0.40
Lower part					
Angle of the piled material—left	(°)	31.77	29.06	36.19	2.15
Angle of the piled material—right	(°)	32.06	29.51	34.94	1.90
Height of the piled material—right	(mm)	7.43	6.76	8.11	0.46
Height of the piled material—middle	(mm)	19.27	16.77	21.00	1.18
Length of the piled material	(mm)	44.14	41.31	45.03	0.94
Discharge time	(s)	2.20	1.94	2.53	0.14
Deflection bar—description	(–)	Increasing height on the deflection plate from bottom to top			
Deflection bar—height	(mm)	3.31	3.72	2.69	0.29

### DEM calibration results

While optimization techniques and surrogate models—such as those proposed by Roessler et al.^24^—can improve efficiency, the manual approach was chosen in this study to ensure direct control over parameter interactions and to establish a validated reference framework.

The virtual silica sand with spherical particles was calibrated using *RR* values of 0.1, 0.15, 0.2, and 0.25. Four sets of calibration simulations were conducted for the spherical particles, totaling 324 simulations. Despite this amount of simulations, it was not possible to identify a suitable set of input parameters that aligned the behavior of the virtual particulate material with experimental results. The results from the initial calibration set (version 1) highlighted that adjusting only the rolling resistance and restitution coefficients was insufficient to replicate the experimental behaviour of the particulate material. This underscored the importance of particle–wall friction (*SF* and *DF*) in influencing flow dynamics within the PFC stand. Describing this initial attempt provides insight into the iterative nature of DEM calibration and supports the rationale for the adjustments made in version 2. Furthermore, documenting version 1 serves to demonstrate the limitations of simplistic parameter combinations, even in large simulation sets. The transition to version 2 was therefore not arbitrary but evidence-based, emerging from observed discrepancies between virtual and experimental results.

Consequently, a new set of simulations (version 2) was created, increasing the friction between the particles and the contact material (*SF* and *DF*). Further 324 simulations were conducted. By adjusting these input friction parameters (*SF* and *DF*), a suitable set of calibration input parameters was successfully achieved. The changed DEM input values of the coefficients of wall friction are displayed in Table 9. These values (*SF* and *DF*) were also applied to polyhedral particles, remaining unchanged from the adjustments made for spherical particles in version 2. Total of 243 calibration simulations were conducted with polyhedral particles using *RR* values of 0.00, 0.05, and 0.10.

**Table 9:** Changed DEM input coefficients of the wall friction.

Wall material	Coefficient of the wall friction (–)
Stainless steel 1.4301	0.65
PMMA	0.64
PLA	0.5

Rolling resistance (*RR*) has a significant impact on the static and dynamic particle behavior. Although DEM contact models for polyhedral particles do not usally consider rolling friction, we applied a small *RR* value to enable direct comparison under consistent calibration conditions. Even minor adjustments to *RR* significantly influenced the behavior of polyhedral particles, highlighting its importance in calibration.

**Fig. 8:**
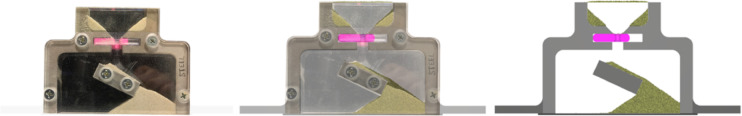
Comparison of the PFC experiment (left), virtual calibration of silica sand behaviour—spherical particles (right), and their overlap (centre).

**Fig. 9:**
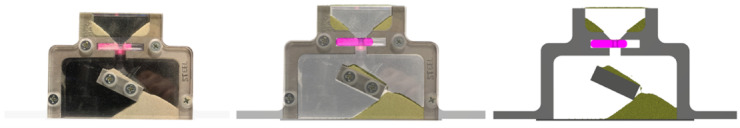
Comparison of the PFC experiment (left), virtual calibration of silica sand KPB behaviour—polyhedral particles (right), and their overlap (centre).

The final calibrated and validated input values for virtual silica sand with spherical particles and polyhedral particles are presented in Table 10, which were based on PFC calibration values showed in Table 11. Comparisons of the powder flow calibration (PFC) experiment, virtual calibration of silica sand behaviour, and their overlap are shown on Fig. 8 (sperical particles) and Fig. 9 (polyhedral particles). It should be noted that the final input DEM parameters were selected as the best set from a range of options. While this range was narrower compared to typical Angle of Repose (AoR) calibration simulations, it was sufficient for these simulations, despite the large number of calibration parameters involved.

**Table 10:** Calibrated and validated input parameters for DEM simulations.

Input parameters	Notation	Spherical particles	Polyhedral particles
Coefficient of restitution	*RC*	0.30	0.70
Static and dynamic friction	*SF and DF*	0.75	0.35
Rolling resistance	*RR*	0.25	0.05

**Table 11:** Values of simulated PFC calibrations.

	Unit	Spheres particles	Polyhedral particles
Upper part			
Angle of the discharged pile—left	(°)	39.71	39.02
Height of the discharged pile—left	(mm)	8.98	9.10
Angle of the discharged pile—right	(°)	39.17	39.33
Height of the discharged pile—right	(mm)	8.61	9.09
Lower part			
Angle of the piled material—left	(°)	35.00	34.79
Angle of the piled material—right	(°)	34.67	33.96
Height of the piled material—right	(mm)	6.77	6.78
Height of the piled material—middle	(mm)	20.85	16.83
Length of the piled material	(mm)	43.43	43.56
Discharge time	(s)	2.45	2.23
Deflection bar—description	(–)	Increasing height on the deflection plate from bottom to top	
Deflection bar—height	(mm)	2.71	3.62

Validation of the virtual material’s behaviour was performed by measuring the discharge time and filling the upper section forming the hopper (as described in Volume Filling Experiment). Both the discharge time and the particle dispersion within the volume confirmed a good match with the selected input parameters of the DEM simulations.

Discharging time in experimental measurements showed considerable variability, with most calibration simulations falling withing this range. Those chosen input parameters sets had their calibration discharge time values within the experimental range (Tables 8 and 11). Similarly, volume-filling validation aligned well with simulation results, though further experiments suggested this consistency may not extend to other materials. Despite this, these validation techniques proved to be sufficiently robust for the material studied.

## Conclusions

We presented the powder flow calibration (PFC) method, applied to over 100,000 spherical and polyhedral particles smaller than 1.5 mm, using dynamic particle behaviour. The study compared spherical and polyhedral particles in DEM calibration for non-cohesive material, revealing key differences. The proposed methodology is based on a novel powder flow calibration (PFC) stand, which combines multiple calibration aspects—discharge time, pile geometry, and dynamic flow behaviour—within a single experimental configuration. The key differences include improved computational power, higher precision compared to the Angle of Repose (AoR) method, increased calibration complexity, the influence of particle shape on material behaviour, and the critical role of rolling resistance (*RR*). Calibration was conducted on silica sand and involved over 1000 simulations using spherical and polyhedral virtual particle shapes.

The powder flow calibration (PFC) stand offers several advantages over traditional rotating drum tests or piling experiments. Unlike piling tests, which primarily focus on static parameters such as the angle of repose, the PFC stand captures dynamic material behaviour, including flow over an obstacle and discharge time. Traditional methods struggle with sub-2 mm particles, whereas the PFC stand was designed specifically for fine particulate calibration. The stand better represents processes such as pneumatic conveying, dosing, feeding, and segregation, which involve material movement rather than just static behaviour. While piling tests or drum tests focus on a few key outputs, the PFC stand captures multiple calibration factors simultaneously, leading to a more robust parameter selection. Thus, the PFC method improves realism and parameter calibration accuracy, refines predictive capabilities, optimizes DEM parameters, and advances knowledge across industries and scientific fields.

This study introduces a methodology for DEM calibration using representative particle shapes rather than attempting to model the full morphological diversity of real particulate systems. Future extensions may include the incorporation of shape libraries which will allow investigation into the sensitivity of calibration outcomes to particle morphology variance.

The calibration process in this study followed an iterative approach. An initial set of 324 simulations (version 1), which varied restitution and rolling resistance parameters, failed to produce a satisfactory match with the experimental results. This outcome demonstrated that adjusting only these parameters was insufficient, particularly in capturing the flow behaviour observed in the PFC stand. Consequently, a second calibration series (version 2) was designed with increased particle–wall friction coefficients (*SF* and *DF*), resulting in significantly improved agreement. This process highlights the importance of considering interaction effects between parameters and validates the need for iterative refinement when calibrating DEM models for fine particles.

Key conclusions from the study include:

- Particle shape plays a critical role: Polyhedral particles yielded more accurate simulation results, especially in dynamic flow regions such as obstacle interactions, although they required significantly more computational time.
- The PFC stand enables multidimensional calibration: Unlike single-output tests such as angle of repose or drum rotation, the PFC method provides nine output variables that allow for robust calibration and cross-validation.
- Manual trial-and-error calibration was employed: Despite the potential of optimization and surrogate models, the calibration relied on a structured manual approach, adjusting *e*_*pp*_, *SF*, *DF*, and *RR* to align simulation with experimental data.
- Version 1 of the calibration (adjusting *e*_*pp*_ and *RR* only) was not sufficient to replicate experimental results, highlighting the importance of including wall friction coefficients (*SF* and *DF*).
- Version 2 of the calibration (adding increased *SF* and *DF*) successfully aligned simulated and experimental behaviours, confirming the need for iterative refinement and interaction-aware tuning.
- The method is applicable in the 50–2000 μm particle size range, constrained by experimental apparatus and simulation stability; outside this range, further investigation will be conducted.

The complexity and time demand of DEM calibration naturally raise the question of whether more efficient procedures could be employed. Instead of trial-and-error simulations, optimization algorithms can efficiently explore the parameter space, e.g.^25^. Some studies use machine learning models trained on experimental datasets to predict DEM parameters with fewer simulations. And lastly, instead of calibrating all parameters simultaneously, a hierarchical calibration approach can be used. We would start with static test calibration of restitution and friction coefficient parameters, then we would approach to dynamic calibration to fine-tune rolling resistance parameters. Final step would be validating the DEM calibration, e.g. by flow behavior or discharge time. However, from our experience, even such a structured approach can sometimes lead to misleading results. Certain interactions between parameters, particularly in non-spherical particle systems, may not be fully captured when calibrating them in isolation. This underscores the importance of validating the final parameter set holistically to ensure that calibration results align with experimental behavior.

Modeling non-spherical particles presents challenges, because they require more computational resources and careful parameter tuning, particularly for particle shape. However, this added complexity yields more realistic behaviour. Finally, here is presented a comprehensive DEM calibration method suitable for cases where upscaling is not feasible. This method is particularly relevant for applications such as mixing, hopper discharge with small outlets, and abrasion, key processes in industries such as mining and process engineering.

## Data Availability

Data availability. Dataset to a research article—Sphere and Polyhedron (project MATUR) (Verze v1) [Data set]. Zenodo. 10.5281/zenodo.14525482.
